# Thermal Conductive Composites Prepared by Addition of Several Ceramic Fillers to Thermally Cationic Curing Cycloaliphatic Epoxy Resins

**DOI:** 10.3390/polym11010138

**Published:** 2019-01-15

**Authors:** Isaac Isarn, Francesco Gamardella, Xavier Fernàndez-Francos, Àngels Serra, Francesc Ferrando

**Affiliations:** 1 Department of Mechanical Engineering, Universitat Rovira i Virgili, C/Av. Països Catalans, 26, 43007 Tarragona, Spain; isaac.isarn@urv.cat; 2 Department of Analytical and Organic Chemistry, Universitat Rovira i Virgili, C/Marcel·lí Domingo s/n, 43007 Tarragona, Spain; francesco.gamardella@urv.cat (F.G.); angels.serra@urv.cat (À.S.); 3 Thermodynamics Laboratory, ETSEIB, Universitat Politècnica de Catalunya, C/Av. Diagonal 647, 08028 Barcelona, Spain; xavier.fernandez@mmt.upc.edu

**Keywords:** cycloaliphatic epoxy resin, composites, thermal conductivity, latency, ceramic fillers

## Abstract

Novel composite coatings prepared from 3,4-epoxy cyclohexylmethyl 3,4-epoxycyclohexane carboxylate (ECC) and different ceramic fillers have been prepared to improve the thermal dissipation of electronic devices. As latent cationic initiator, a benzylanilinium salt with triethanolamine has been used, which leads to a polyether matrix. Different proportions of Al_2_O_3_, AlN and SiC as fillers were added to the reactive formulation. The effect of the fillers selected and their proportions on the evolution of the curing was studied by calorimetry and rheometry. The thermal conductivity, thermal stability, thermal expansion coefficient and thermomechanical and mechanical properties of the composites were evaluated. An improvement of 820% in thermal conductivity in reference to the neat material was reached with a 75 wt % of AlN, whereas glass transition temperatures higher than 200 °C were determined in all the composites.

## 1. Introduction

Epoxy thermosets are widely used in electric and electronic industries and other engineering applications. Usually, they are applied as adhesives or protective coatings, due to their good processability before curing, good compatibility with a large number of materials, high electrical insulating characteristics, and good thermal, corrosion and chemical resistance [1]. However, unmodified epoxy thermosets show low thermal conductivity, typically around 0.2 W/m·K, which may limit their use when thermal dissipation is required [2]. It must be noticed, that the dissipation of heat generated becomes a non-ignorable issue in the miniaturization of electronic devices. The increasing temperature, as the result of the generated heat in specific parts of the devices, greatly affects the performance of the electronic equipment, leading to malfunction, poor reliability, and premature failures. The dissipation of heat is usually done by applying a heat sink, for example, a metal plate, and a cooling fan to blow away local heat. In order to keep good thermal contact between heat sources and heat sinks and to improve heat transfer efficiency, materials with high thermal conductivity, but electrically insulating, are needed to fill the gaps [3].

One of the easiest methods to solve the inherent low conductivity of epoxy resins, with the lowest cost and effectiveness, is to introduce fillers with high thermal conductivity combined with electrical insulating properties. Among them, boron nitride (BN) is one of the most used fillers, which combines properties such as high thermal conductivity, high dielectric strength, low thermal expansion coefficient (CTE) and low density [4,5]. However, some authors reported the use of other fillers to get those improvements [6]. Among them, ceramics such as aluminum oxide (Al_2_O_3_) [7] and aluminum nitride (AlN) [8], silicon nitride (Si_3_N_4_) [9], silicon carbide (SiC) [10] and graphene oxide (GO) [11] are ideal candidates in terms of their wide band gap and high thermal conductivity [3].

In the present paper, we have selected three of them: aluminum oxide and nitride and silicon carbide and they are added as fillers in cycloaliphatic epoxy resin formulations. Al_2_O_3_ particles are widely used in the electronic field, because of their cheaper price compared to other ceramic fillers, despite their relatively lower thermal conductivity (38–42 W/m·K) [12]. Its CTE is reported to be 7 ppm/°C. AlN particles are relatively expensive, but show higher thermal conductivity (150–220 W/m·K) and lower thermal expansion coefficient (CTE) (2.5–5 ppm/°C) [12]. Finally, SiC has an intermediate thermal conductivity of 85 W/m·K [12] and CTE on the range 4.1–4.7 ppm/°C [3]. With regards to the cycloaliphatic epoxy, few studies are based on this. The combination of small and compact structure and high oxirane content gives resins with low viscosity and thermosets with high weatherability, low dielectric constant, in addition to high T_g_ [1]. This class of epoxy is popular for diverse end uses including auto topcoats, weatherable high-voltage insulators, ultraviolet (UV) coatings and encapsulants for electronic and optoelectronic applications, and in the last times there is an interest in growing applications including lithographic inks and photoresists for the electronics industry [13]. The inherent low viscosity of these resins enables them to be formulated with higher levels of inorganic fillers. This enhances mechanical and electrical track resistance for electrical and electronic components [14]. In the present work, we have used as thermal curing agent an ammonium salt (CXC1612) with a small proportion of triethanolamine (TEA) that confers a latent character to the curing system. In a previous work, it has been proved its latency and the fact that it leads to thermosets with high glass transition temperature (T_g_) [15].

By the use of different fillers, this study aims at reaching better results than those obtained in a previous study in which BN was used [15]. In that study, an increase of 800% in thermal conductivity was obtained by adding 40 wt % of BN to the formulation. The materials obtained presented T_g_ values higher than 200 °C, reductions in CTE up to 50% in reference to the neat epoxy material and improved shear strength and microindentation hardness. The addition of BN filler did not compromise the latency of the curing system, due to their inert character in this polymerization mechanism. The different chemical characteristics of the fillers selected in the present study could affect the curing kinetics, and it is foreseeable they greatly influence the thermal and mechanical characteristics of the final composites.

## 2. Materials and Methods 

### 2.1. Materials

3,4-epoxy cyclohexylmethyl 3,4-epoxycyclohexane carboxylate (ECC) (ERL-421D, EEW = 126.15 g/eq) was provided by Dow Chemical Company (Midland, MI, USA). Initiator CXC1612 from King Industries Inc., Norwalk, CT, USA, which was determined to be *N*-(4-methoxybenzyl)-*N*,*N*-dimethylanilinium hexafluoroantimonate, was dissolved in propylene carbonate at 50 wt %. Propylene carbonate and triethanolamine (TEA) were provided by Sigma-Aldrich (Darmstadt, Germany) and purified by distillation. Aluminum oxide (Al_2_O_3_) from Showa Denko (Tokyo, Japan) with a particle size of 1–5 µm (ρ = 3.95 g/cm^3^), aluminum nitride (AlN) from Sigma Aldrich (Darmstadt, Germany) with a particle size of 0.5–3 µm (ρ = 3.26 g/cm^3^) and silicon carbide (SiC) 1D 99/CM101/F1200 from Shengli Abrasives (Dongying, China) with approximate size of 4–8 µm (ρ = 3.21 g/cm^3^).

### 2.2. Sample Preparation

The mixtures were prepared by mixing ECC with 1 phr of CXC1612 (parts of initiator per hundred parts of resin) and 0.1 phr of TEA. For composite samples, the required amount of filler was added in wt % to the previous formulation. The mixtures were stirred mechanically until homogeneity and degassed under vacuum to prevent the appearance of bubbles during the curing process. Finally, the samples were poured onto aluminum molds coated with Teflon and cured following a multi-step temperature schedule at 100, 120, 150, 180 and 200 °C, with a dwelling time of 1 h at each temperature.

### 2.3. Characterization Techniques

Differential scanning calorimetry (DSC, Mettler Toledo, Columbus, OH, USA) was used to study the curing evolution by using a Mettler DSC-821e calibrated using an In standard (heat flow calibration) and an In-Pb-Zn standard (T calibration). Samples of approx. 5–10 mg were tested in aluminum pans with a pierced lid in N_2_ atmosphere with a gas flow of 100 mL/min. The dynamic studies were performed between 30–250 °C with a heating rate of 10 °C/min. The reaction enthalpy (*Δh*) was integrated from the calorimetric heat flow signal (*dh/dt*) using a straight baseline, with the help of the STARe software.

Rheometric measurements were carried out in a TA Instruments AR G2 rheometer (New Castle, DE, USA), equipped with electrical heated plates (EHP) with parallel plate geometry (25 mm diameter disposable aluminum plates). Complex viscosity (η*) and viscoelastic properties of the mixtures prepared were recorded as function of angular frequency *ω* (rad/s) in the linear range of viscoelasticity, obtained from constant shear storage modulus (*G’*) in a strain sweep experiment at 1 Hz at 30 °C.

Thermal stability of cured samples was evaluated in a Mettler thermogravimetric analysis/simultaneous difference thermal analysis (TGA/SDTA) 851e thermobalance (Columbus, OH, USA). All the experiments were carried out under N_2_ atmosphere (100 mL/min). Pieces of cured samples of 5–10 mg were heated between 30 and 600 °C at a heating rate of 10 °C/min. Dynamic mechanical thermal analyses (DMTA) were performed by using a TA Instruments DMA Q800 analyzer (New Castle, DE, USA). Prismatic rectangular samples (15 × 6 × 2.3 mm^3^) were analyzed by 3-point bending at a heating rate of 3 °C/min from 35 to 300 °C using a frequency of 1 Hz and oscillation of 0.1% of sample deformation. The Young’s modulus (*E*) was determined at 30 °C in a controlled force experiment using a three point bending clamp, as explained in a previous paper [4]. Thermomechanical analyses (TMA) were carried out on a Mettler TMA40 thermomechanical analyzer (Columbus, OH, USA). Cured samples (12 × 12 × 2.3 mm^3^) were supported by the clamp and one silica disc to distribute uniformly the force and heated at 5 °C/min from 35 to 100 °C by application of 0.01 N, a minimum force to avoid distortion of the results. 

Knoop microindentation hardness was measured as reported before with a Wilson Wolpert 401 MVA (Microhardness Vickers Analog) device following ASTM D1474-13 (Wolpert Wilson Instruments, Aachen, Germany). 20 determinations were made for each material to reach a confidence level of 95%. Surface fracture was examined with a FEI Quanta 600 environmental scanning electron microscope (ESEM, FEI Company, Hillsboro, OR, USA) that allows collecting electron micrographs at 10–20 kV and low vacuum mode of uncoated specimens with low electron conductivity. 

Thermal conductivity was measured using the Transient Hot Bridge method by a THB 100 device from Linseis Messgeräte GmbH (Selb, Germany). A HTP G 9161 sensor was used with a 3 × 3 mm^2^ area calibrated with poly(methyl methacrylate) (PMMA), borosilicate crown glass, marble, Ti-Al alloy and titanium. Two equal polished rectangular samples (12 × 12 × 2.3 mm^3^) were placed in each one of the faces of the sensor. Due to the small size of the sensor, side effects can be neglected. A measuring time of 100 s with a current of 10 mA was applied to each of the five measures done for the different formulations. 

## 3. Results and Discussion

### 3.1. Calorimetric Study of the Curing Process

**Figure 1:**
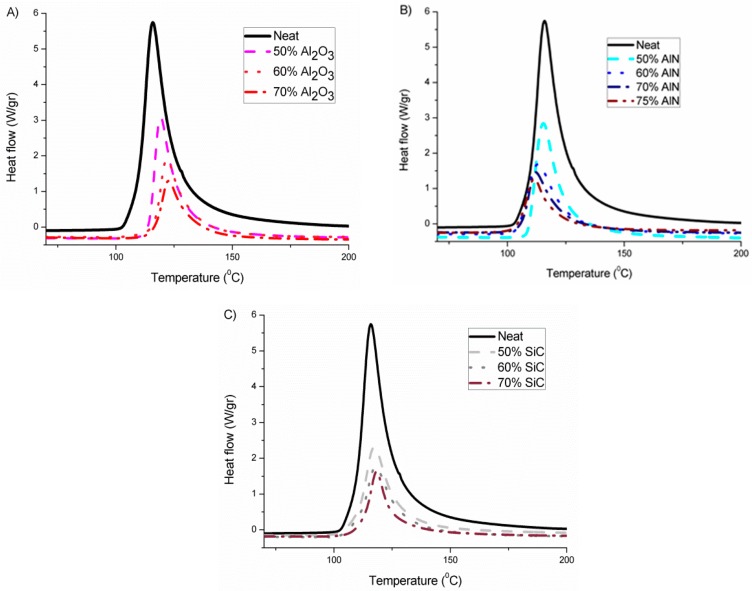
Exothermic curves from differential scanning calorimetry (DSC) analysis of all the mixtures prepared with different fillers: (**A**) Al_2_O_3_; (**B**) AlN; (**C**) SiC.

To ascertain the influence of the different fillers selected in the curing evolution, proportions of 50, 60 and 70 wt % of each type of particle fillers were added to the epoxy resin/initiator formulations and studied by DSC. In the case of AlN, a higher proportion (75 wt %) was added after examining the rheological results obtained. As the curing system, we selected a thermal cationic latent epoxy system previously reported that consist in *N*-(4-methoxybenzyl)-*N*,*N*-dimethylanilinium hexafluoroantimonate with a small proportion of triethanolamine that leads to the homopolymerization of the cycloaliphatic epoxy in a latent manner [15]. Figure 1 presents the non-isothermal calorimetric curves of the different formulations studied, where we can see the different effects for the fillers selected. 

In Figure 1A, it can be seen that the addition of alumina produces a delay in the curing process on increasing its proportion in the formulation, which can be related to its basic character that interacts with the growing cationic species, deactivating them. By contrast, AlN shows in Figure 1B a small acceleration of the reaction which is accentuated on rising its concentration. Thus, it seems that AlN structure favors the formation and stability of the cations. The addition of the third filler, SiC in Figure 1C, leads to practically the same peak temperature as the neat epoxy, which means that it has an inert character in the reaction mechanism and that no interaction between the cationic species formed and the filler structure occurs. The interaction between growing polymer chains and fillers can also play a role in the thermal transmission, because in the interphase phonons are usually scattered, reducing the heat transfer.

The most representative data obtained from the calorimetric analysis are collected in Table 1.

In addition to the onset and the temperature of the maximum of the peak, the heat evolved by gram or by epoxy equivalent was calculated. This value gives us valuable information about the conversion of epoxide groups achieved in all the composites prepared. The value of enthalpy should approach the one of the neat formulation. As we can see, the heat released in the neat formulation is the highest, which indicates that the addition of filler to the formulation reduces the conversion achieved, probably due to topological restrictions produced by the particles. However, the addition of alumina leads to the lowest enthalpy released, which can be related to the inactivation of the growing cationic species in the basic alumina surface. In previous studies with BN as the filler no effect was observed in the kinetics which agrees with the inert chemical character of the BN particles, but there was a reduction in the released enthalpy, independent of the filler content, related to topological hindrance to the homopolymerization reaction [15]. All the cured samples were submitted to a second DSC scan but no T_g_ could be observed due to the high crosslinking achieved in the epoxy matrix because of the compact structure of the epoxy resin and the homopolymerization produced in the curing.

### 3.2. Rheological Behavior of Mixtures

**Figure 2:**
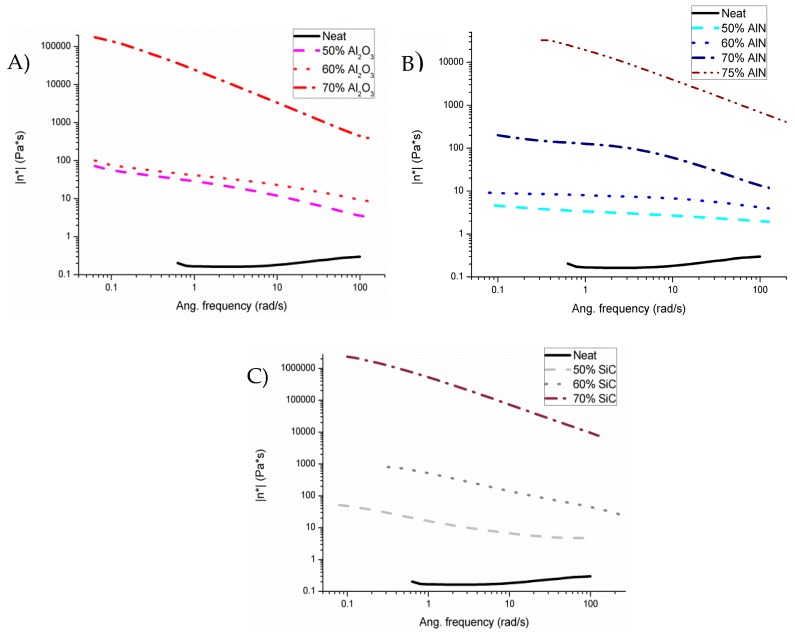
Complex viscosities of mixtures varying frequency in the linear viscoelastic region (LVR) of each formulation at 30 °C with different fillers: (**A**) Al_2_O_3_; (**B**) AlN; (**C**) SiC.

Mixtures before curing were essayed by rheology to analyze their viscoelastic behavior. The particle size of the different fillers is in the same range, and the particle shapes are quite similar. These two properties are the ones having the strongest influence on the rheological behavior. Although density differences in the fillers selected lead to a variation on their volumetric content, the similarity between particles allowed us to perform the comparison among the different formulations [16,17]. The linear viscoelastic region (LVR) was determined in oscillatory tests, varying amplitude of deformation with a fixed frequency (1 Hz) at 30 °C. As in previous studies with BN [4,15,18], the LVR is displaced to lower amplitudes when the filler proportion increases. This means that the mixture’s microstructure finds a critical strain above which the structure organization starts to breakdown at lower amplitudes when the amount of filler grows [19]. Thus, the amplitude was set at the LVR for each mixture to carry out the frequency sweep experiments. The effect of the filler content for each type of filler on the complex viscosity (η*) is represented in Figure 2.

As we can see in all the mixtures, to a greater or lesser extent, shear thinning is observed. This is due to a diminution of viscosity on increasing the frequency applied, attributable to changes in the microstructure of the mixture, typical of filled blends. In contrast, the neat formulation represents an almost constant value on varying the frequency, which means that it has a Newtonian behavior. Mixtures with alumina and silicon carbide present a very large difference in viscosity between 60 and 70 wt % (3 orders of magnitude) and 5–7 orders of magnitude in reference to the neat formulation. On the other hand, the addition of aluminum nitride does not produce any large effect until the addition of 75 wt %. Thus, from the point of view of the application on surfaces or filling molds, aluminum nitride is the best suited. 

**Figure 3:**
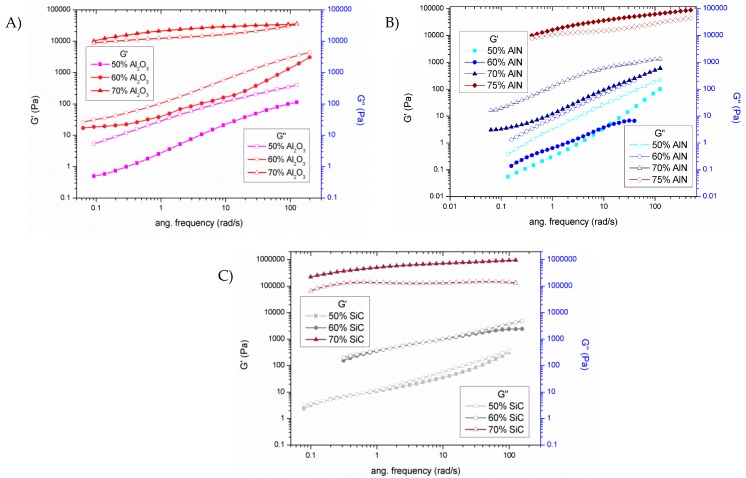
Plots of G’ and G’’ versus frequency in frequency sweep tests at 30 °C for the different formulations studied with different fillers: (**A**) Al_2_O_3_; (**B**) AlN; (**C**) SiC.

Studies on micro and nanoparticle composites showed that the thermal conductivity of the composites is significantly smaller than that of their bulk counterparts due to phonon-interface scattering [20]. Commonly, the used fabrication techniques, such as hot pressing, tend to produce randomly particle distributed composites, forming clusters of particles. When particles with high thermal conductivity are randomly dispersed in a matrix material with low transport properties, the largest cluster can form a percolation network [21]. Thus, the largest cluster of thermally conductive material can connect the opposite boundaries when the volumetric concentration of particles reaches certain limit. This limit of volumetric concentration, defined as the percolation threshold, is determined by the geometric characteristics of particles. The percolating network can create a low resistance pathway for thermal transport and the conductivity of the composites can increase notably once reached percolation [22]. The significant changes in the microstructure, related to the percolation threshold, can be evaluated by the study of storage modulus (G’) and loss modulus (G’’), which determine the elastic and viscous properties, respectively. Figure 3 shows the plots of both moduli on changing the frequency for all the formulations studied.

An interpretation of Rouse-like behavior takes as a criterion that when G’ and G’’ at low frequencies become equal the percolation threshold has been reached [18,23]. This means the transition from non-percolated to percolated response must be accompanied by the change from the liquid-like behavior (G’ < G’’) to solid-like behavior (G’ > G’’). In Figure 3A this change is observed between the mixtures of 60 and 70 wt % of alumina, as corresponds to the large increase in viscosity (Figure 2A). In case of AlN blends (Figure 3B) only liquid like behavior is exhibited until the 70% of filler added. For this reason, a new mixture with the 75% of AlN was also prepared to surpass the rheological percolation. It must be commented, that this is the practical limit for a hand-mixing procedure. Since all particle sizes are similar, the lower viscosity of AlN formulations could be attributed to the lower specific surface area (SSA) of AlN powder. Finally, the mixtures with 50 and 60 wt % of SiC (Figure 3C) are very close to reach the percolation but this is not surpassed until 70% wt % of SiC. A high increase of viscosity is observed in the transition (Figure 2C). Apart from the high volumetric ratio due to its low density, this high viscosity is assumed to be related to the high SSA.

### 3.3. Morphological Analysis

**Figure 4:**
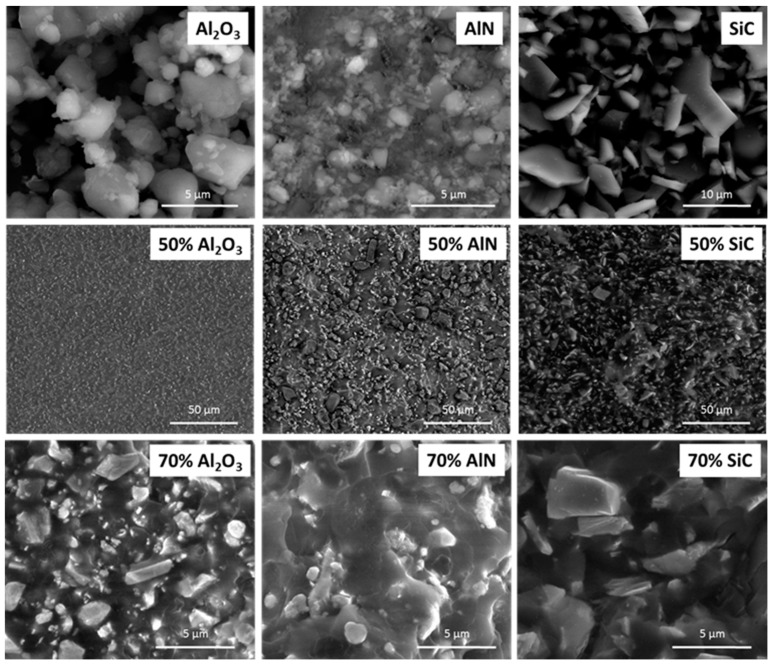
Environmental scanning electron microscope (ESEM) micrographs of fillers and fracture surfaces of composites at different magnifications.

The appearance of the different fillers used in this study as well as the fracture surface of the prepared composites was analyzed by ESEM. Representative micrographs are shown in Figure 4. 

The morphological characteristics of the fillers used in the present study are shown in the first row of the figure. In the next two rows, two images of each type of composite with different filler contents and different magnifications have been also included. As we can see, SiC is formed by particles with a polyhedral shape with smooth surface. In the composite with a 70 wt %, these particles are not well bonded to the polymeric matrix, which agrees with the inert character of SiC in the DSC kinetic study. The particles are very close to each other, which confirms that the percolation has been reached.

The particles of alumina and AlN are like aggregates and polydisperse in size. However, AlN particles are much smaller and more polydisperse. Both type of particles seems to be quite well bonded to the epoxy matrix as can be seen in the micrographs of the bottom row, which accounts for an interaction of the surface groups with the growing chains, as was deduced from the retardation and acceleration of the curing process detected by calorimetric studies. The micrograph of AlN at 70 wt % composite show that some of the particles are isolated, since at this filler content percolation has not been reached. The inspection of the fracture surfaces of the samples with a 50 wt % of each filler allows confirming the homogeneous distribution of the particles on the epoxy matrix and the tough characteristics of the fracture, since the fracture cracks are very tortuous. 

### 3.4. Thermal and Mechanical Characterization of Composites

The addition of filler can increase some thermomechanical properties of polymeric materials. Particles act as a reinforcement of the polymer network leading to the improvement of some properties as thermal stability, stiffness or hardness. 

TGA, DMTA, TMA and microindentation experiments were performed in the composites prepared to evaluate these characteristics and to investigate the effect of each of the fillers on them. 

**Figure 5:**
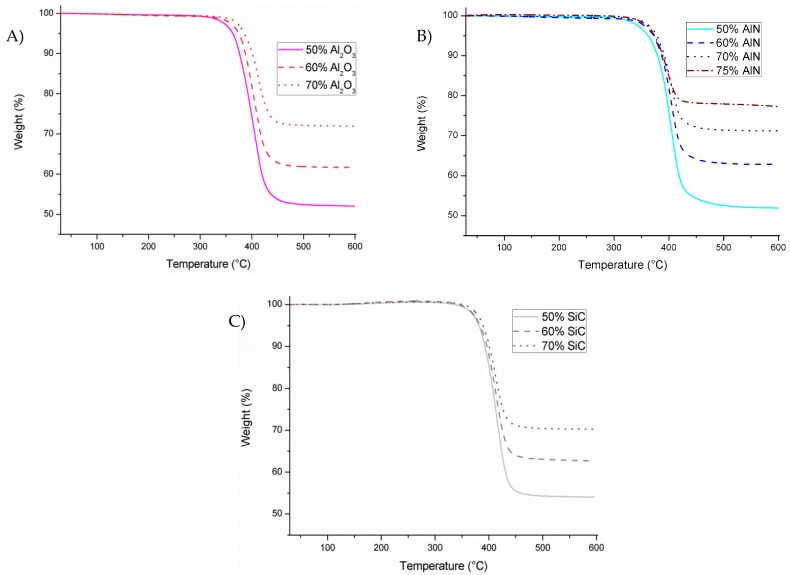
Thermogravimetric analysis (TGA) degradation curves of the different composites obtained by the addition of with different fillers: (**A**) Al_2_O_3_; (**B**) AlN; (**C**) SiC.

The evaluation of the thermal stability performed by TGA analysis is represented in Figure 5. As can be seen, the shape of the curves is similar which implies that the presence of fillers does not influence the degradation mechanism.

Table 2 collects the data of char yields and the temperature of 2% of decomposition (T_2%_). A large difference can be observed in the T_2%_ between the neat epoxy and the composites, of around 100 °C. In a previous study [15] using the same epoxy system but adding BN till 40 wt % this increase was more than 70 °C. The reason for this increase is the less resin content to be degraded and therefore T_2%_ occurs at higher temperature. However, on changing the type of filler these temperatures experiment small variations when the same proportion was added, which could be attributed to differences in the organic network structure. In fact, greater differences on changing the proportion are observable for alumina and AlN composites, in line with the effect of the filler on the curing process observed with DSC. As can be seen in the table, practically the total amount of ECC is decomposed in inert atmosphere and char residues agrees quite well with the proportion of filler added. What can be assumed is that the introduction of particles does not negatively affect the thermal stability of composites.

**Figure 6:**
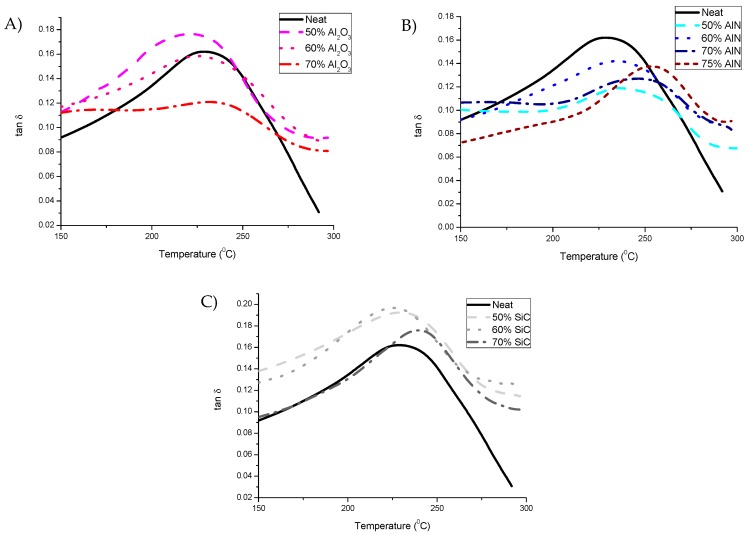
Curves of tan δ for the composites prepared determined by DMTA. (**A**) Al_2_O_3_; (**B**) AlN; (**C**) SiC.

Thermomechanical behavior of composites was analyzed by DMTA. Table 2 shows the rigidity and the temperature of the maximum of tan δ peak, related to T_g_. Young’s modulus measured at 30 °C is highly increased with the addition of filler. This is due to the mechanical reinforcement role played by the particles within the matrix. In Figure 6, the tanδ curves are shown. 

It can be seen that the relaxation curves are very broad and have low intensity as in ECC-BN composites [15], indicating a slow relaxation process and a low homogeneity in the network structure due the high crosslinking density and to the inherent inhomogeneity caused by ring-opening polymerization mechanism. As DMTA evaluates the thermomechanical characteristics, the results are influenced by the mechanical role played by the particles on the organic matrix. In this way, it can be seen a notable change in the T_g_ of the materials with alumina and SiC when it exceeds percolation. Conversely, materials with AlN show a gradual increase of T_g_ without major changes when percolation is achieved. 

The values of the storage modulus (E’) in the rubber state could not be determined, since in the tan δ curves we can observe that the complete relaxation of the material is higher than 300 °C, and at this temperature the decomposition of the polymeric matrix has already begun. 

The thermal expansion coefficient is an important parameter for the reliability and working life of epoxy materials when they operate as coatings or as thermal interface materials (TIMs) since changes in their working temperature can produce high internal stresses due to the mismatch between the CTE the epoxy coating or TIM, and the substrate, the former being larger [24]. This can provoke de-adhesion between the interfaces they bond, wrapping or any type of defects that finally reduce the durability of the materials. Reducing the CTE of the epoxy coating or TIM would reduce the CTE mismatch and this would result in an enlargement of the working-life of the devices.

CTEs were evaluated by TMA and the results are given in Table 2. As expected, on increasing the filler content CTE’s are notably reduced. The reduction of this coefficient is gradual with the percentage of filler added. The CTE of the ceramic filler does not affect much the values of the composite materials. However, the addition of 75 wt % of AlN, which has the lowest CTE, leads to the lowest value of 22 ppm/K in all the composites prepared.

**Figure 7:**
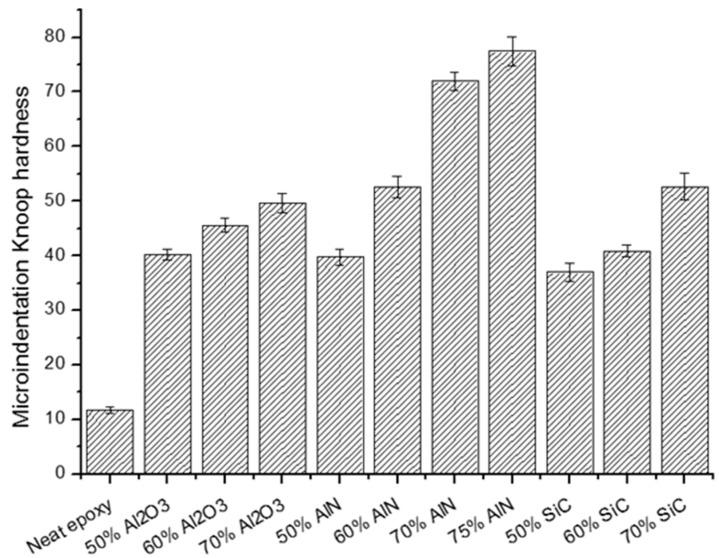
Microindentation Knoop hardness dependence on filler/proportion in the composites.

Another characteristic that has an effect on the durability of composites is the surface hardness of the materials. By means of microindentation tests, the Knoop hardness of each composite was determined and the values are represented in Figure 7. 

As in the case of materials with BN [4,15,18], the hardness increased with the filler loading. The change of the type of particles added should also affect in principle, the improvement reached. It is reported that the hardness of the ceramic fillers follows the order SiC > Al_2_O_3_ > AlN [25]. However, this trend is not followed in our composites, since the greater effect on hardness is originated by AlN, which leads to more than 6-fold improvements on adding a 75 wt % of this filler to the epoxy formulation. These unexpected results could be rationalized in a better interaction between the organic matrix with the filler in case of AlN, or to the particle size and shape that undoubtedly should affect hardness characteristics. In a previous study on composites with the same polymer matrix but a 40 wt % BN filler, the maximum hardness value reached was only 26.6 KHN (Knoop Hardness) and the progressive improvement of the hardness with the filler content occurs more smoothly than in the present study [15]. However, in that work the proportion of filler was lower, the percolation was reached at 14.4 wt %, because of the platelet-like shape of the particles. This leads to the conclusion that for hardness improvement, the fillers used in the present study are better than the previous studied h-BN filler, and among them, AlN is the most effective.

### 3.5. Thermal Conductivities

**Figure 8:**
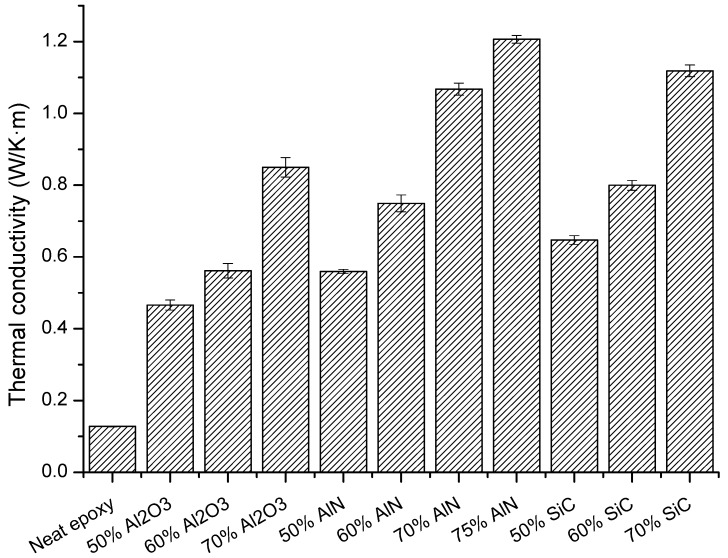
Thermal conductivities of the neat epoxy and the different composites prepared.

The main goal of the study was to increase the thermal conductivity of the cycloaliphatic epoxy matrix by the addition of thermal conductive particles. The results of the thermal conductivity determined for the composites prepared are represented in Figure 8.

The highest conductivity values (1.21 W/m·K) were reached for the sample with a 75 wt % of AlN, followed by the composite with the highest proportion of SiC (1.12 W/m·K). However, it is quite curious that at the same filler content (70%) SiC leads to a higher value of this parameter, although pure AlN has a much higher conductivity than pure SiC. This unexpected result could be explained on the basis of the conductive pathways formed by the filler particles, which is related to the percolation phenomenon, which for AlN has not been achieved at this filler content. 

The thermal conductivity obtained in the present study is higher than that obtained in BN composites (1.04 W/m·K) [15]. However, in the present case a higher proportion of filler has been added to the formulation, since the addition of h-BN was limited to 40 wt %, because of the difficulty of the manual preparation of mixtures with a higher proportion of this filler. However, the different shape and size of the filler particles finally leads to big differences in the surface area and on the interactions with the organic matrix and makes it difficult to come to general conclusions. In a previous study, we could demonstrate the influence of the size and shape of the particles on the thermal conductivity of this type of composites [18].

As we have seen, the higher the proportion of filler, the better is the thermal conductivity, but any big difference is observed between the different particles used, being the most important effect the achievement of the percolation. However, it should be taken into account that mechanical properties and processability can become worse with a high proportion of fillers and both characteristics should also be considered in the improvement of thermal management. For this reason, as an alternative, the use of nanofillers, with high specific surface area that can be chemically modified, will be attempted in the forthcoming studies of our group.

## 4. Conclusions

The addition of different ceramic fillers to an epoxy cycloaliphatic formulation cured with a latent cationic homopolymerization system led to an acceleration on adding AlN, a delay in the case of alumina, whereas the addition of SiC did not produce any kinetic effect in the curing process. The addition of filler slightly reduced the degree of curing, probably due to topological restrictions. The use of alumina as the filler led to the lowest curing degree, which could be related to the basic character of alumina.

The viscosities of the filled formulations increased significantly on reaching the percolation. The addition of alumina or SiC in a proportion of 70 wt % surpassed the percolation threshold but it is necessary to reach a 75 wt % of AlN to experiment with this phenomenon.

The different fillers did not affect the thermal degradation mechanism of the composites but the initial degradation temperature increased in 100 °C in the highest loaded composites in reference to the neat material. 

The materials presented a broad relaxation curve in the DMTA, according to their high crosslinked structure. T_g_ values increased with the filler content.

Young’s moduli and hardness were enhanced significantly by the addition of the reinforcements. The maximum values were reached in the 75 wt % of AlN composite with a 6-fold and a 7-fold increase, respectively, in reference to the neat material. This material showed the lowest CTE value of all the materials prepared.

As a general conclusion, it can be stated that the formulation with a 75 wt % of AlN is the most adequate in terms of applicability (lower viscosity) and the material obtained after curing has the best mechanical performance, the highest T_g_, the lowest thermal expansion coefficient, and the highest thermal conductivity.

## Figures and Tables

**Table 1:** DSC data of the different formulations studied.

Sample	T_onset_^a^ (°C)	T_peak_^a^ (°C)	*Δh*^b^ (J/g)	*Δh*^b^ (kJ/ee)
Neat	109	117	596	75.2
50% Al_2_O_3_	114	120	258	65.2
60% Al_2_O_3_	116	122	202	63.8
70% Al_2_O_3_	117	123	147	62.0
50% AlN	109	116	286	72.2
60% AlN	106	113	225	71.0
70% AlN	106	112	172	72.1
75% AlN	106	111	146	73.4
50% SiC	109	118	277	69.9
60% SiC	110	118	221	69.8
70% SiC	112	120	166	69.6

^a^ Onset and maximum peak temperatures of the curing exotherm. ^b^ Heat evolved during the curing by gram of mixture or by epoxy equivalent.

**Table 2:** Thermogravimetric and thermomechanical data from the composites prepared.

Sample (wt %)	Vol. Fraction (%)	T_2%_^a^ (°C)	Char Yield ^b^ (%)	E ^c^ (GPa)	T_g_^d^ (°C)	CTE ^e^ (10^−6^·K^−1^)
Neat epoxy	-	273	1.0	2.4	227	115
50% Al_2_O_3_	22.8	341	52.0	6.1	223	58
60% Al_2_O_3_	30.8	354	61.6	7.5	223	38
70% Al_2_O_3_	40.9	368	71.9	11.1	244	36
50% AlN	26.4	337	51.9	7.1	235	56
60% AlN	35.0	351	62.8	7.8	238	38
70% AlN	45.6	359	71.2	12.2	246	35
75% AlN	51.8	353	77.3	14.4	252	22
50% SiC	26.6	368	54.0	7.8	230	52
60% SiC	35.3	370	62.7	10.4	230	48
70% SiC	46.0	377	72.6	11.6	240	32

^a^ Temperature of 2% weight loss determined by TGA in N_2_ at 10 °C/min. ^b^ Char residue at 600 °C (in N_2_). ^c^ Young’s modulus determined by dynamic mechanical thermal analyses (DMTA) at 30 °C. ^d^ Temperature of maximum of tanδ at 1 Hz in a DMTA oscillatory experiment using the same clamp. ^e^ Thermal expansion coefficient in the glassy state determined between 50–75 °C by thermomechanical analyses (TMA).
